# Cocaine- and Amphetamine-Regulated Transcript (CART) Signaling within the Paraventricular Thalamus Modulates Cocaine-Seeking Behaviour

**DOI:** 10.1371/journal.pone.0012980

**Published:** 2010-09-23

**Authors:** Morgan H. James, Janine L. Charnley, Emma Jones, Emily M. Levi, Jiann Wei Yeoh, Jamie R. Flynn, Douglas W. Smith, Christopher V. Dayas

**Affiliations:** Neurobiology of Addiction Laboratory, School of Biomedical Sciences and Pharmacy and the Centre for Brain and Mental Health Research, University of Newcastle and the Hunter Medical Research Institute, Newcastle, New South Wales, Australia; Chiba University Center for Forensic Mental Health, Japan

## Abstract

**Background:**

Cocaine- and amphetamine-regulated transcript (CART) has been demonstrated to play a role in regulating the rewarding and reinforcing effects of various drugs of abuse. A recent study demonstrated that i.c.v. administration of CART negatively modulates reinstatement of alcohol seeking, however, the site(s) of action remains unclear. We investigated the paraventricular thalamus (PVT) as a potential site of relapse-relevant CART signaling, as this region is known to receive dense innervation from CART-containing hypothalamic cells and to project to a number of regions known to be involved in mediating reinstatement, including the nucleus accumbens (NAC), medial prefrontal cortex (mPFC) and basolateral amygdala (BLA).

**Methodology/Principal Findings:**

Male rats were trained to self-administer cocaine before being extinguished to a set criterion. One day following extinction, animals received intra-PVT infusions of saline, tetrodotoxin (TTX; 2.5 ng), CART (0.625 µg or 2.5 µg) or no injection, followed by a cocaine prime (10 mg/kg, i.p.). Animals were then tested under extinction conditions for one hour. Treatment with either TTX or CART resulted in a significant attenuation of drug-seeking behaviour following cocaine-prime, with the 2.5 µg dose of CART having the greatest effect. This effect was specific to the PVT region, as misplaced injections of both TTX and CART resulted in responding that was identical to controls.

**Conclusions/Significance:**

We show for the first time that CART signaling within the PVT acts to inhibit drug-primed reinstatement of cocaine seeking behaviour, presumably by negatively modulating PVT efferents that are important for drug seeking, including the NAC, mPFC and BLA. In this way, we identify a possible target for future pharmacological interventions designed to suppress drug seeking.

## Introduction

Cocaine- and amphetamine-regulated transcript (CART) is a neuropeptide that was originally identified in the striatum of animals following acute psychostimulant exposure [1]. Subsequent studies have identified that CART is expressed in a number of regions known to be involved in reward and reinforcement, including the ventral tegmental area (VTA), nucleus accumbens (NAC), amygdala and hypothalamus [2], [3]. Early studies firmly established a role for CART in appetite control, with central administration of CART found to dose-dependently suppress feeding behaviour [4]. More recently however, there has been a renewed interest in the role that CART might play in modulating the rewarding and reinforcing effects of drugs of abuse, and in particular, psychostimulants such as cocaine and amphetamine [5]–[10].

The effects of CART on the reinforcing and locomotor-activating properties of psychostimulants are complex and appear to be both brain region- and dose- dependent. For example, administration of the active CART55-102 peptide into the VTA results in a cocaine-like increase in locomotor activity and produces a conditioned place preference (CPP) similar to that induced by cocaine or amphetamine [11], indicating that CART signaling in this region is reinforcing. However, administration of CART55-102 into the NAC or the ventral pallidum (VP) significantly attenuates the locomotor effects of acute cocaine and amphetamine administration [12]–[14], and prevents the expression of conditioned hyperlocomotion in a cocaine-paired environment [15]. Together, these data suggest that CART signaling within the NAC and VP works to negatively regulate the effects of psychostimulants. Interestingly, when administered into the basolateral amygdala (BLA), lower doses of CART appear to be rewarding, whilst higher doses are aversive [6]. Taken together, these findings strongly implicate CART as a regulator of the reinforcing and rewarding effects of psychostimulants, however the effects appear to be highly regional and dose specific.

A recent study suggests that CART may also be involved in mediating the reinstatement of extinguished drug seeking, as intracerebroventricular (i.c.v.) administration of CART was found to prevent context-induced reinstatement of alcohol seeking in rats [16]. Importantly though, it remains unclear as to whether CART mediates reinstatement in animals that have a significant history of psychostimulant exposure. Further, it is important that the site(s) at which CART exerts its inhibitory effects in a reinstatement model be determined.

A region of particular interest with regards to the integration of CART peptide activity is the paraventricular thalamus (PVT). The PVT is a part of the midline and intralaminar thalamic group and is known to receive dense innervation from CART-containing neurons in the lateral hypothalamus [17]–[19]. Importantly, the PVT is known to project to a number of regions implicated in the reinstatement of drug seeking, including the NAC, medial PFC (mPFC) and BLA [18], [20]–[23] and therefore represents a possible site through which hypothalamic CART activity may be relayed to relapse-relevant regions. Consistent with this idea, presentation of stimuli previously associated with alcohol availability increases the activation of neurons within the PVT and these neurons are closely apposed to CART terminal fields [10]. Further, excitotoxic lesions of the PVT have been shown to prevent context-induced reinstatement of alcohol seeking [24]. At present however, it is unclear whether the role of the PVT extends to other forms of reinstatement (e.g. drug-primed reinstatement) and to other drugs of abuse (e.g. psychostimulants). Further, it is unclear whether any role that the PVT may play in reinstatement is directly related to CART signaling in this region.

Therefore, here we examined the role that the PVT plays in modulating drug-primed reinstatement of cocaine seeking by functionally inactivating the PVT with the sodium channel blocker tetrodotoxin (TTX) prior to reinstatement testing. We also investigated the effects of intra-PVT infusions of the active CART55-102 neuropeptide on reinstatement behaviour. We found that treatment with either TTX or CART produced a significant attenuation of drug-seeking behaviour following cocaine prime.

## Results

### Guide Cannulae Placement

**Figure 1:**
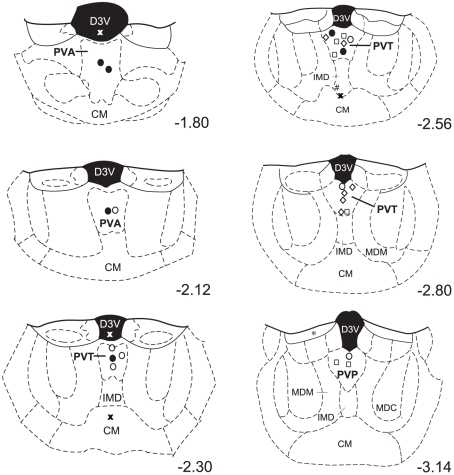
Location of microinfusion injector tips. The majority of injections were made directly into the PVT region. Four TTX-treated animals and two CART-treated animals had injections that fell beyond the PVT boundary, and the responding of these animals was assessed to determine the specificity of the observed effects to the PVT. Numbers represent the approximate rostrocaudal distance from bregma. Figures adapted from Paxinos and Watson [44]. Symbols represent different groups; •: saline; ○: TTX; ◊: 0.625 µg CART; □: 2.5 µg CART; X: misplaced TTX; *: misplaced 0.625 µg CART injections; #: misplaced 2.5 µg CART injections. CM: central medial thalamic nucleus; D3V: dorsal third ventricle; IMD: intermediodorsal thalamic nucleus; MDC: mediodorsal thalamic nucleus, central part; MDM: mediodorsal thalamic nucleus, medial part; PVA: Paraventricular thalamic nucleus, anterior part; PVT: Paraventricular thalamic nucleus; PVP: Paraventricular thalamic nucleus, posterior part.

As shown in Figure 1, guide cannulae from the majority of animals were localized to the PVT. Four TTX-treated animals were found to have guide cannulae that were misplaced; two were directed at the third ventricle, another at the central medial thalamic nucleus (CM), and the other at the boundary of the intermediodorsal thalamic nucleus (IMD) and the CM. The guide cannulae from one animal treated with 0.625 µg CART was directed at the stria medullaris of the thalamus, whilst one animal treated with 2.5 µg CART had its guide cannulae directed at the lateral boundary of the IMD and the CM.

### Self-Administration Training and Extinction

Rats developed stable responding (±10% over 3 sessions) for cocaine within 8 days of training (±1.04 SEM). Over the last three days of cocaine self-administration training, the mean number of cocaine infusions per session across all animals was 29.32 (±1.43 SEM), which equated to approximately 19 mg/kg of cocaine per session. Importantly, levels of cocaine consumption did not differ across the four treatment groups. During cocaine-self administration, animals significantly favoured the active cocaine-paired lever as compared to the inactive lever (*F*_1,27_ = 130.84, *p*<.001) and this preference did not differ between the treatment groups (*F*_3,27_ = 1.47, *p* = .24). Animals met the extinction criteria after an average of 20.65 (±1.87 SEM) days and the number of days taken to reach the extinction criterion was indistinguishable across all groups (*F*_3,30_ = .95, *p* = .43).

### Reinstatement Testing

**Figure 2:**
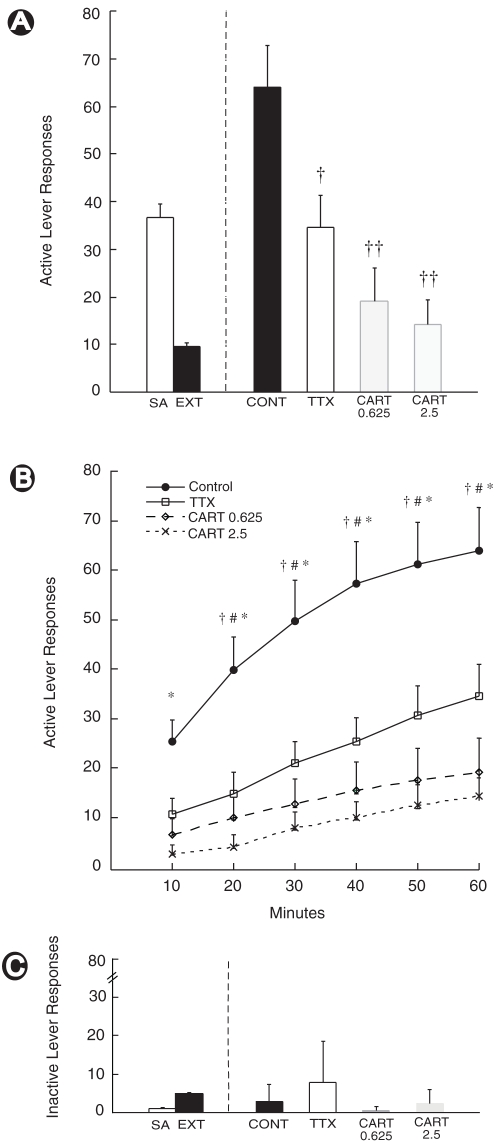
Microinfusion of TTX or CART into the PVT attenuates cocaine-primed reinstatement. All animals were tested following a cocaine prime (10 mg/kg, i.p.) preceded by a PVT-directed microinfusion of either saline, 2.5 ng TTX, 0.625 µg CART55-102 or 2.5 µg CART55-102 (*n* = 6–7). A group of animals served as no-injection controls, and were grouped with saline-treated animals to form a ‘control’ group (CONT). Treatment with TTX or CART produced a significant attenuation of active lever responding following cocaine prime, as compared to controls †**:** *p* = .05, ††: *p*<.01, significantly different to controls (A). Control animals exhibited a significantly higher level of active lever responding than 2.5 µg CART-treated animals in the first ten minutes of reinstatement testing. At all other time points, control animals exhibited significantly higher levels of responding than TTX- and both CART-treatment groups. †: Control group significantly different to TTX-treatment group; #: Control group significantly different to 0.625 µg CART-treatment group; * : Control group significantly different to 2.5 µg CART-treatment group, all *p*s<.05 (B). Importantly, TTX and CART treatment did not affect responding on the inactive lever, indicating that the observed effects were specific to drug-seeking behaviour rather than an overall reduction in arousal and/or locomotion (C). SA: Average number of active lever presses over last three days of self-administration training period. EXT: Average number of active lever presses over last three days of extinction training. Error bars represent (+ S.E.M.).

After reaching the extinction criterion, all animals were subjected to a cocaine prime. Animals that received intra-PVT saline-injections prior to cocaine-priming injections displayed an identical reinstatement of responding on the active lever to animals that were prepared with PVT-guide cannulae but received no injection (*F*_1,11_ = .60, *p* = .46). We therefore combined these two groups to form a single ‘Control’ group (*n* = 12). ANOVA revealed a significant main effect of ‘session’ (*F*_1,27_ = 30.24, *p*<.001), indicating that the cocaine-prime brought about an overall increase in responding from extinction levels. A significant ‘session’ x ‘treatment’ interaction was also observed (*F*_3, 27_ = 8.26, *p*<.001) suggesting differences in the extent to which the treatments affected cocaine prime-induced reinstatement of responding. Planned, separate post-hoc analyses revealed that TTX-treated animals displayed on average a lower level of responding on the active lever compared to controls (*p* = .05). Similarly, animals treated with either 0.625 µg CART55-102 and 2.5 µg CART55-102 exhibited significantly reduced reinstatement responding compared to controls (*p*s<.01), with the 2.5 µg CART dose having the greatest effect (see Figure 2A). Importantly, changes in lever pressing behaviour by all treatments was limited to the active lever with neither a significant main effect (*F*_1,27_ = 1.97, *p* = .17) nor a significant interaction (*F*_3,27_ = 1.47, *p* = .25) observed for left (inactive) lever responses during the test phase (see Figure 2C).

We also assessed differences in responding on the active lever at ten-minute intervals across the one-hour reinstatement session (see Figure 2B). Repeated measures ANOVA revealed a significant ‘time’ x ‘treatment’ interaction (F_675.63, 137.70_ = 4.91, *p*<.01, Huynh-Feldt correction). Planned post-hoc t-test comparisons revealed that the control group exhibited significantly greater levels of responding in the first ten minutes of reinstatement testing than the 2.5 µg CART group (*p*<.05, adjusted for multiple comparisons), but not the TTX- or 0.625 µg CART- treated groups. At all other time points, the control group exhibited significantly greater levels of responding than all other treatment groups (*p*s<.05, adjusted for multiple comparisons).

We also examined the responding of animals that had misplaced guide cannuale to determine the specificity of the observed effects to the PVT. The four animals that received misplaced TTX injections (*M* = 49.25, *SE* = 27.86) did not differ in their active lever responses to the control group (*M* = 64, *SE* = 8.69; *F*_1,15_ = 0.47, *p* = .50), but did respond on average at a higher rate to animals that received PVT-directed TTX (*M* = 34.57, *SE* = 6.25). Similarly, the two animals that received misplaced CART injections (*M* = 64.5, *SE* = 22.5) responded on the active lever at a level almost identical to controls, but higher than the PVT-directed 0.625 µg CART (*M* = 19, *SE* = 6.47) and 2.5 µg CART (*M* = 14.17, *SE* = 4.73) groups. As such, the effect of both TTX and CART treatment was most pronounced when injected into the PVT.

## Discussion

The purpose of the present study was two-fold. Firstly, we aimed to investigate whether the role of the PVT in modulating reinstatement to cue and context-induced alcohol seeking extended to drug-primed cocaine seeking. To do this, we used TTX administration to functionally inactivate the PVT prior to cocaine-primed reinstatement testing. We report that TTX-treated animals exhibited significantly attenuated drug-seeking behaviour following drug prime, showing for the first time that the PVT plays an important role in mediating drug primed ‘relapse’ of cocaine-seeking behaviour. Our second aim was to determine whether CART signaling within the PVT might regulate drug-seeking behaviour, as we have previously shown CART-containing terminals within the PVT to be closely apposed to drug-cue activated PVT neurons [10]. Further, PVT cells project to a number of relapse-relevant brain regions including the NAC, mPFC and BLA [18]–[23]. To achieve these aims, animals were treated with either 0.625 µg or 2.5 µg of the CART55-102 peptide, based on previous studies that examined the role of CART in drug-motivated behaviours [6], [11], [13], [15], [16]. CART produced a significant attenuation of drug-seeking following drug-prime, with the 2.5 µg dose having the greatest effect. These findings build upon previous reports that i.c.v. CART inhibits reinstatement [16] by identifying a specific site at which CART acts to modulate relapse-like behaviour.

It is noteworthy that PVT-directed treatment did not alter responding on the inactive lever, suggesting that the effects we observed were specific to drug-seeking rather than a generalized reduction in arousal or locomotor activity as a result of CART or TTX infusion. Whilst it is possible that low levels of overall responding on the inactive lever may have masked a non-specific effect of CART or TTX, several pieces of evidence indicate that this is highly unlikely. Firstly, previous work studying the effects of ibotenic acid lesions of the PVT on renewal of beer seeking reported no alterations in inactive nose-poke responding [24]. Presumably, these lesions also disrupted CART signaling onto PVT cells as well as the actions of other inputs into this site. Secondly, i.c.v. [16] and intra-accumbal infusions [15] of CART resulted in a specific reduction of drug-related locomotor activity, and i.c.v. CART injections do not alter spontaneous locomotor activity [4]. It is also important to note that CART has been reported to produce some postural changes and motor tremors. For example, i.c.v. infusion of 1 µg or 2 µg of CART produced a modest movement-associated tremor, however this did not impair the ability of the animals to engage in reward-seeking behaviour, nor did it significantly alter locomotor activity [4]. Motor tremors were also observed following intra-VTA infusion of a 5 µg dose of CART, but not a 1 µg dose [11]. Importantly, close observation of our animals revealed no evidence of motor effects at the relatively low doses of CART used in our study.

It should also be noted that a dorsal approach to the PVT for the placement of guide cannulae was employed in our study. Whilst this approach, as compared to an angled approach, may have marginally increased the possibility of the injectate spreading dorsally into the D3V, we are confident that the observed effects were specific to the PVT region. This conclusion is based on the responding seen from animals with misplaced injections of TTX directed at the D3V that resulted in responding that was indistinguishable from control animals. Similarly, animals that received TTX and CART injections that were directed at thalamic structures surrounding the PVT and, indeed one case that was adjacent to the D3V, also exhibited responding that was identical to controls, again indicating PVT-specific effects. Importantly, CART and TTX infusions were made at various rostro-caudal levels of the PVT, and responding within each treatment group was consistent at all injection sites. This is consistent with anatomical tracing studies that suggest that the number of projections to relapse relevant brain regions is relatively consistent along the entire rostro-caudal length of the PVT [18].

The PVT is known to respond to noxious stimulation, and inputs from tactile and nociceptive pathways have been hypothesised to be integrated within intralaminar and midline thalamic nuclei [25], [26]. Further, hypothalamic-pituitary-adrenal (HPA) axis [27] and central amygdala [28] responses to stress are altered by functional manipulations of the PVT. It has been inferred from these data that the PVT plays an important role in arousal and attentional control. The present findings build upon this literature and strongly support an emerging body of evidence that suggests that the PVT also plays a critical role in reward-related processing [29]. For example, PVT lesions blocked the conditioned locomotor response to a cocaine-paired environment in a sensitization experiment [30], whilst PVT activation has been shown to be associated with increased locomotor responses entrained to regulated feeding schedules [31]. Particularly pertinent to the present study, the PVT has recently been implicated in modulating the reinstatement of alcohol seeking. For example, presentation of cues previously associated with alcohol availability increased c-fos expression within the PVT [10], whilst ibotenic acid lesions of the PVT prevented context-induced reinstatement of alcohol seeking [24]. Together, these data indicate a role for the PVT in reward-seeking behavior that we demonstrate here also includes reinstatement of drug-seeking behaviour precipitated by a cocaine prime.

It has recently been suggested that the PVT modulates reward-related behaviour by acting to integrate and relay hypothalamic activity to reward-relevant regions, including the NAC and perhaps the mPFC [29]. Indeed, anatomical tracing studies have shown that the PVT receives dense innervation from the lateral hypothalamus [18], [19], [32], [33] and in turn sends glutamatergic projections to the NAC, mPFC and BLA [20]–[23]. Further, stimulation of the PVT has been shown to modulate neuronal excitability in both the NAC and PFC [23], [34], [35]. Interestingly, a significant percentage of PVT neurons send collateral (i.e. branched) projections to both the NAC and mPFC, suggesting that the PVT is anatomically positioned to simultaneously influence both of these drug-relevant regions [20], [22]. Furthermore, CART-positive terminal fibres in the PVT are closely apposed to neurons that project directly to the NAC shell [19]. Taken together with the data presented here, we propose that CART signaling within the PVT acts to inhibit drug-primed reinstatement by modulating the activity of PVT-efferents, including to the NAC and/or mPFC. Indeed, electrophysiological studies indicate that CART can produce inhibitory post-synaptic effects when applied to brain slices [36].

Consistent with this suggestion, anatomical studies show that glutamatergic efferents from the PVT are closely associated with dopamine immunoreactive terminals in the NAC shell, and this relationship may potentiate NAC activity [37]. Indeed, stimulation of the PVT increases dopamine efflux within the shell of the NAC [35]. The NAC has been extensively demonstrated to be critically involved in the reinstatement of drug-seeking behaviour. For example, NAC shell inactivation suppresses reinstatement of drug-seeking elicited by contextual cues [38] and NAC shell neurons show increased levels of activation after presentation of drug-paired discriminative stimuli measured using Fos [39] or single unit recordings [40]. In addition, dopamine antagonist injections aimed at the NAC shell block the reinstatement of cocaine seeking induced by a drug prime [41]. Taken together, these data indicate that CART might act to suppress drug-primed reinstatement by preventing PVT-glutamate efferents modulating the responsivity of NAC neurons to cocaine-induced dopamine release.

In summary, we demonstrate that the PVT plays an integral role in modulating drug-primed reinstatement of cocaine seeking and that CART signaling within this region negatively regulates relapse-like behaviour. Whilst not directly addressed here, CART signaling in the PVT may act to negatively modulate dopamine release within the NAC and other regions known to be involved in reinstatement. As such, CART signaling within the PVT may represent a potential target for pharmacological interventions designed to suppress drug-seeking behaviour. It will be for future studies to determine if CART signaling also suppresses drug seeking evoked by cues linked to drug taking and stress. Presumably however, a full evaluation of the potential role of CART as a therapeutic treatment target awaits the identification of the receptor system through which this peptide acts.

## Materials and Methods

### Ethics Statement

All procedures performed were approved by the University of Newcastle Animal Care and Ethics Committee (approval number 1068), and were carried out in accordance with the New South Wales Animal Research Act.

### Animals

Male Sprague-Dawley rats (Central Animal House, University of Newcastle, NSW, AUS; weighing 200–250 g upon arrival) were housed two per cage on a reverse 12-hour light/dark cycle (lights off at 7:00 am) with *ad libitum* access to food and water.

### Drugs

Rat CART55-102 (Phoenix Pharmaceuticals, CA, USA) and TTX (Alomone, Israel) were dissolved in sterile saline and stored at 4°C until use. Both doses of CART55-102, TTX, and saline were microinjected at volumes of 0.25 µl.

### Surgery

#### Catheterisation

Prior to surgery, rats were injected intramuscularly with 0.3 mL of a broad-spectrum antibiotic (150 mg/mL procaine penicillin, 112.5 mg/mL benzathine penicillin; Norbrook Laboratories, UK) and subcutaneously with 0.2 mL of a 50 mg/mL solution of carprofen (Norbrook Laboratories, UK). Rats (250–300 g) were anesthetized with isofluorane (1–3% with a flow rate of 2 L/min) and, using aseptic procedures, a Silastic catheter was surgically implanted into the right jugular vein as described in detail previously [42]. Post-surgery, jugular catheter lines were flushed with 0.3 mL of 50 mg/mL cephazolin (Mayne Pharma, Australia). To ensure catheter patency throughout the course of the experiment, catheters were flushed daily with 0.2 mL of 50 unit heparinised saline.

#### PVT-Directed Guide Cannulae

Prior to surgery, animals were treated with procaine penicillin and carprofen as above. Animals were anesthetized with isofluorane before being placed in a sterotaxic frame (Stoelting, IL, USA). Craniotomies were made into the skull to facilitate the insertion of four stainless steel jeweller's screws (Mann Optics, QLD, AUS), whilst a fifth craniotomy was made to allow the insertion of a stainless steel guide cannula (26 guage, Small Parts, FL, USA) to the level of the PVT (−2.6 mm AP relative to bregma, −4.6 mm DV relative to skull surface). Guide cannulae were secured to the four screws with dental cement (Henry Schein, AUS). Cannulae were kept clear by using stainless steel stylets (33 guage, Small Parts, FL, USA) of identical length to the guides.

### Behavioural Training

Behavioural procedures were conducted in standard operant conditioning chambers located inside sound-attenuating, ventilated cubicles (Med Associates, VT, USA). Chambers were equipped with two retractable levers (6 cm above the floor), white cue lights (one above each lever), two speakers to deliver auditory stimuli and a house light located at the top of the chamber wall opposing the levers. A syringe pump (5 rpm motor, Med Associates, VT, USA) located on the outside of the sound-attenuating cubicle delivered the IV cocaine and saline vehicle solutions. Data acquisition and behavioural testing equipment were controlled by a Windows-based PC, using MED-PC IV (Med Associates, VT, USA).

Seven days after surgery, rats were trained to self-administer cocaine hydrochloride (Johnson Matthey, Edinburgh, UK; dissolved in sterile physiological saline 2.5 mg/mL) intravenously in three-hour sessions conducted daily for 5 days a week. Responding on the active (right) lever resulted in a 4 second infusion of cocaine (0.1 mL) via the intravenous catheter and activation of a white cue light above the active lever that remained illuminated for 20 seconds signaling a time out period. The inactive (left) lever was not extended during the initial training sessions. Training was continued until stable responding for cocaine was achieved (±10% rewarded responses over 3 sessions) at which time animals were subjected to eight two-hour sessions whereby both the active (right) and inactive (left) levers were presented. In these sessions, responding on the active lever had similar consequences as in the initial training phase, whilst responding on the inactive lever was recorded but had no scheduled consequences.

Three days following the final training session, animals were implanted with PVT-directed guide cannulae and were allowed one week for recovery. Lever responding was then extinguished by again presenting both the active and inactive levers, but with IV infusions withheld. Extinction trials continued until a criterion of ≤10 responses per session on the active lever over three consecutive days was achieved.

### Reinstatement Testing

Over the final three to five days of extinction training, animals were gently restrained whilst their stylet was removed and replaced, in order to condition animals to the testing procedure. One day following the final extinction session, animals were again restrained whilst their stylet was removed and placed in 70% ethanol. Animals then received a microinfusion of either saline (*n* = 6), 2.5 ng TTX (*n* = 11), 0.625 µg CART (*n* = 7) or 2.5 µg CART (*n* = 7). A group of six animals were implanted with guide cannulae into the PVT but were not subjected to intra-PVT injections and served to control for the effect of intra-PVT drug infusion on cocaine prime-induced responding. Based on previous studies the selected dose of TTX was expected to inactivate tissue within a ∼0.35–0.4 mm radius from the site of injection [43] and therefore have minimal effect on structures surrounding the PVT. Further, the doses of CART were selected based on previous reports that these doses are sufficient to modulate the reinforcing effects of cocaine [13], [15]. All microinjections were made through an injector cannulae (30 guage, Small Parts, FL, USA), which protruded 1.3 mm below the tip of the guide cannula into the PVT region. Infusions were delivered using a Hamilton microsyringe mounted on a motorised pump (Stoelting, IL, USA) at a rate of 2 µl/min. The injector remained in the guide cannulae for a further minute following administration in order to allow adequate diffusion of the injectate into the PVT. Animals were then administered a cocaine drug-prime (10 mg/kg, i.p.) and placed into the operant chamber. Testing for reinstatement of responding began five minutes later under extinction conditions for one hour. Two hours following commencement of testing, animals were deeply anaesthetized with sodium pentobarbital (200 mg/kg IP) and transcardially perfused with 150 mLs of PBS (pH 7.4) followed by 500 mLs of 4% paraformaldehyde (pH 9.5) to allow for verification of injector sites.

### Data Analysis

Separate one-factor ANOVAs were used to compare the experimental and control groups in terms of number of training days, overall cocaine intake, time taken to reach extinction, and responding on both the active and inactive levers over the last three days of extinction. Lever preference during training was assessed using a 2 ‘Lever’ (active, inactive) ×4 ‘Treatment’ (Control, TTX, 0.625 µg CART, 2.5 µg CART) mixed-model ANOVA, whilst reinstatement of responding on both the active and inactive levers was analysed using a 2 ‘Session’ (extinction, reinstatement) ×4 ‘Treatment’ (Control, TTX, 0.625 µg CART, 2.5 µg CART) mixed-model ANOVA. Where significant interactions were observed, planned Tukey post-hoc tests were used to assess differences between treatment groups. Differences in active lever responding at various timepoints across the one hour reinstatement session was assessed using a 4 ‘Treatment’ (Control, TTX, 0.625 µg CART, 2.5 µg CART) X 6 ‘Time’ (10 min, 20 min, 30 min, 40 min, 50 min, 60 min) repeated measures ANOVA. Where significant interactions were observed, planned t-test analyses were used to compare treatment groups and Bonferroni α corrections were made to control for family-wise error. An alpha level of .05 was used for all statistical tests.
